# Simultaneous Production of Multiple Antimicrobial Compounds by *Bacillus velezensis* ML122-2 Isolated From Assam Tea Leaf [*Camellia sinensis* var. *assamica* (J.W.Mast.) Kitam.]

**DOI:** 10.3389/fmicb.2021.789362

**Published:** 2021-11-24

**Authors:** Patthanasak Rungsirivanich, Elvina Parlindungan, Paula M. O’Connor, Des Field, Jennifer Mahony, Narumol Thongwai, Douwe van Sinderen

**Affiliations:** ^1^Department of Biology, Faculty of Science, Chiang Mai University, Chiang Mai, Thailand; ^2^Graduate School, Chiang Mai University, Chiang Mai, Thailand; ^3^School of Microbiology, University College Cork, Cork, Ireland; ^4^APC Microbiome Ireland, University College Cork, Cork, Ireland; ^5^Teagasc Food Research Centre, Moorepark, Fermoy, Ireland; ^6^Research Center in Bioresources for Agriculture, Industry and Medicine, Chiang Mai University, Chiang Mai, Thailand

**Keywords:** amylocyclicin, bacteriocin, biocontrol, gene cluster, Miang, plipastatin, RT-qPCR, surfactin

## Abstract

*Bacillus velezensis* ML122-2 is an antimicrobial-producing strain isolated from the leaf of Assam tea or Miang [*Camellia sinensis* var. *assamica* (J.W.Mast.) Kitam.]. The cell-free supernatant (CFS) of strain ML122-2 exhibits a broad-spectrum antimicrobial activity against various Gram-positive and Gram-negative bacteria as well as the mold *Penicillium expansum*. The genome of *B. velezensis* ML122-2 was sequenced and *in silico* analysis identified three potential bacteriocin-associated gene clusters, that is, those involved in the production of mersacidin, amylocyclicin, and LCI. Furthermore, six gene clusters exhibiting homology (75–100% DNA sequence identity) to those associated with the secondary metabolites bacilysin, bacillibactin, surfactin, macrolactin H, bacillaene, and plipastatin were identified. Individual antimicrobial activities produced by *B. velezensis* ML122-2 were purified and characterized by Matrix-assisted laser desorption ionization-time of flight (MALDI-TOF) mass spectrometry analysis, revealing three antimicrobial peptides with molecular masses corresponding to surfactin, plipastatin, and amylocyclicin. Transcriptional analysis of specific genes associated with mersacidin (*mrsA*), amylocyclicin (*acnA*), plipastatin (*ppsA*), and surfactin (*srfAA*) production by *B. velezensis* ML122-2 showed that the first was not transcribed under the conditions tested, while the latter three were consistent with the presence of the associated peptides as determined by mass spectrometry analysis. These findings demonstrate that *B. velezensis* ML122-2 has the genetic capacity to produce a wide range of antimicrobial activities that may support a specific community structure and highlight the biotechnological properties of Assam tea.

## Introduction

Members of the *Bacillus* genus, which represent Gram-positive and endospore-forming bacteria, are widespread in a variety of environments including air, soil, aquatic ecosystems, foods, skin, and the gastrointestinal tract of animals (Abriouel et al., 2011). Some species of *Bacillus* are believed to play a key role in biological control through the production of antimicrobial compounds (e.g., bacteriocins, non-ribosomal polypeptides, and polyketides) and in plant growth promotion, such as *Bacillus amyloliquefaciens*, *Bacillus subtilis*, and *Bacillus tequilensis* (Chen et al., 2007; Gao et al., 2017; Li et al., 2018). The antimicrobial metabolites produced by *Bacillus* spp. are used in clinical settings to achieve inhibition of pathogens, such as *Bacillus cereus*, *Clostridium difficile*, *Listeria monocytogenes*, and methicillin-resistant *Staphylococcus aureus* (MRSA; Sabaté and Audisio, 2013; Lv et al., 2020; Rungsirivanich and Thongwai, 2020). Additionally, certain species of *Bacillus* have been reported to elicit probiotic potential, in particular *Bacillus amyloliquefaciens*, *Bacillus licheniformis*, *Bacillus pumilus*, *Bacillus siamensis*, and *Bacillus subtilis* (Du et al., 2018; Jeżewska-Frąckowiak et al., 2019; Rungsirivanich et al., 2020).

Bacteriocins are ribosomally synthesized antimicrobial peptides which exhibit antimicrobial activity mostly against closely related bacterial species (Klaenhammer, 1993). Bacteriocins have been classified into three major classes: Class I bacteriocins are small peptides which undergo post-translational modifications, while Classes II and III are small (0.77–10kDa) and large (>10kDa) unmodified linear antimicrobial proteins, respectively (Abriouel et al., 2011; Cotter et al., 2013; Alvarez-Sieiro et al., 2016). In addition to bacteriocins, several species of *Bacillus* have been described to produce non-ribosomally synthesized peptides (NRPs) and polyketides (PKs) with antimicrobial properties (Patel et al., 1995; Pathak and Keharia, 2013). NRPs and PKs are synthesized by large multi-modular synthetases, non-ribosomal peptide synthetases (NRPSs), polyketide synthetases (PKSs), or hybrid NRPS/PKS enzymes. NRPSs typically consist of one or more modules, each responsible for the enzymatic incorporation of a specific amino acid in a growing peptide. An individual NRPS module typically consists of three core domains, that is, domains responsible for adenylation, thiolation, and condensation. Similarly, a given PKS enzyme comprises acyl transferase, acyl carrier, and ketosynthase domains (Mootz et al., 2002; Wang et al., 2014; Aleti et al., 2015). Prediction of gene clusters responsible for the biosynthesis of antimicrobial compounds using genome-mining tools has been applied for the identification and subsequent characterization of genes associated with antimicrobial compound production (Medema et al., 2011; Sekurova et al., 2019). BAGEL is a powerful prediction tool aimed at the identification of bacteriocin-associated genes (De Jong et al., 2006). AntiSMASH is a genome database for gene cluster analysis responsible for the synthesis of secondary metabolite compounds, such as NRPs, PKs, and other antimicrobials (Medema et al., 2011; Weber et al., 2015).

Matrix-assisted laser desorption ionization-time of flight mass spectrometry (MALDI-TOF MS) is an analytical technique used for evaluating chemical components which are ionized into charged molecules. It has been applied for identification and analysis of biological molecules, especially proteins and peptides (Singhal et al., 2015). MALDI-TOF MS has also been used to identify and analyze antimicrobial peptides, such as amylocyclicin (Scholz et al., 2014), iturin, fengycin, surfactin (Yang et al., 2015; Théatre et al., 2021), and mersacidin (Viel et al., 2021).

Antimicrobial peptides (AMPs) have received substantial attention as an effective treatment of bacterial infections and as an alternative to antibiotics (Cotter et al., 2005), in many cases supported by their low toxicity to human cells (Yang et al., 2014). Furthermore, specific AMPs have not only been used in the food industry as preservatives but also in agricultural applications as antimicrobial compounds (Dischinger et al., 2014). Several studies have identified antimicrobial-producing *Bacillus* strains associated with soils and plants and are therefore believed to contribute to the biocontrol of plant pathogens (Shafi et al., 2017; Andrić et al., 2020). The mode of action of bacteriocins may be through interaction with specific membrane receptors causing bacterial membrane disruption and associated electrolyte leakage from bacterial cells, ultimately leading to cell death (Tymoszewska et al., 2017; Perez et al., 2018). In contrast, antibiotics typically act as enzyme inhibitors in DNA replication, protein, and fatty acid synthesis, or cell wall biosynthesis (Zhang et al., 2018; O’Rourke et al., 2020). Previous studies by Rungsirivanich and Thongwai (2020) and Rungsirivanich et al. (2020) revealed the antimicrobial activity of *B. velezensis* ML122-2 isolated Assam tea [*Camellia sinensis* var. *assamica* (J.W.Mast.) Kitam.] leaf surface against *S. aureus*, including MRSA. Moreover, this strain was also shown to exhibit tannin tolerance and probiotic properties. In the current study, we describe the identification, purification, and characterization of antimicrobial compounds produced by *B. velezensis* ML122-2 revealing co-production of several distinct antimicrobial compounds. Genome and transcriptional analysis of *B. velezensis* ML122-2 revealed expression of the corresponding gene clusters for these antimicrobial activities.

## Materials and Methods

### Bacterial Strain and Growth Condition

*B. velezensis* ML122-2 (previously named *Bacillus siamensis* ML122-2; GenBank accession no. MH796212) was isolated from an Assam tea leaf [*Camellia sinensis* var. *assamica* (J.W.Mast.) Kitam.] harvested in the Phrae province, Thailand. Strain ML122-2 was grown in tryptic soy broth (TSB, Merck^™^, Germany) at 37°C with shaking at 150rpm for 24h, as previously described by Rungsirivanich and Thongwai (2020).

### Antimicrobial Activity Assay

Antibacterial activity was assayed using an agar well diffusion method according to the modified protocol of Sewify et al. (2017). The indicator bacteria (listed in Table 1) were grown in brain heart infusion (BHI, Oxoid^™^, Basingstoke, England), de Man, Rogosa, and Sharpe (MRS, Oxoid^™^, Basingstoke, England), or M17 (Oxoid^™^, Basingstoke, England) containing 0.5% (w/v) glucose (GM17) broth for pathogenic, lactic acid bacteria (LAB), and *Floricoccus penangensis* ML061-4, respectively, prior to incubation at 37°C (for pathogenic bacteria) or 30°C (for LAB) overnight. Each culture broth was adjusted to a turbidity equivalent of 0.5 McFarland standard. 100μl of an indicator culture was spread onto the agar surface. The agar diffusion assay was also utilized to evaluate the antifungal potential of *B. velezensis* ML122-2 employing a method adapted from Yang and Chang (2010). Fungal strains *Penicillium digitatum* DSM 2731 and *Penicillium expansum* DSM 1282 were obtained from the DSMZ culture collection (Braunschweig, Germany) and were cultivated on Sabouraud 4% dextrose agar (Sigma-Aldrich^™^, St. Louis, MO, United States) at 30°C for at least four days or until sporulation occurred. Fungal spore suspensions were prepared by scraping spores from the surface of the mold lawn and suspending the spores in ^1^/_4_ strength Ringer’s solution containing 0.8% Tween 80. Approximately 10^4^ to 10^5^ spores/ml were seeded into Sabouraud 4% dextrose semi-solid agar. The agar was punctured using a sterile tip to make a hole with an 8mm diameter. 100μl of *B. velezensis* ML122-2 filtrate was incorporated into each well, after which plates were incubated at the appropriate temperature for 24–48h. Antimicrobial activity, as observed by a clear (due to lack of fungal growth) zone around the well, was measured in millimeters of clearing zone diameter.

**Table 1:** Antimicrobial activity of cell-free supernatant (CFS) produced by *B. velezensis* ML122-2 against indicator microorganisms.

Microorganism	Zone of inhibition (mm)
*Bacillus cereus* TISTR 687^a^	10.1±0.2
*Bacillus subtilis* NCDO 1769^b^	0
*Bacillus subtilis* NCDO 10073^b^	0
*Enterobacter aerogenes* NCIMB 10102^c^	9.0±0.0
*Escherichia coli* DH5α^d^	0
*Floricoccus penangensis* ML061-4^e^	11.3±0.3
*Lactococcus lactis* HP^d^	13.9±0.9
*Leuconostoc paramesenteroides* NCDO 1012^b^	12.0±0.0
*Leuconostoc paramesenteroides* NCDO 869^b^	16.5±0.0
*Levilactobacillus brevis* MB 521^d^	14.5±0.8
*Levilactobacillus brevis* SA-C12^d^	15.3±0.8
*Levilactobacillus brevis* Rap 51^d^	0
*Levilactobacillus brevis* Rap 43^d^	13.1±0.4
*Levilactobacillus brevis* 56^d^	14.9±0.2
*Levilactobacillus brevis* ATCC 347^f^	14.8±0.3
*Listeria innocua* UCC3^d^	11.1±0.2
Methicillin-resistant *Staphylococcus aureus* DMST 20625^g^	10.4±0.4
*Penicillum digitatum* DSM 2731^h^	0
*Penicillum expansum* DSM 1282^h^	29.8±0.8
*Pseudomonas aeruginosa* PA 01^d^	10.1±0.2
*Staphylococcus aureus* ATCC 25923^f^	10.4±0.4
*Staphylococcus aureus* NCDO 947^b^	9.6±0.6
*Streptococcus dysgalactiae* grp B^d^	10.3±0.4
*Weissella cibaria* R16^d^	11.9±0.2
*Weissella confusa* I5^d^	12.0±0.4

Data are expressed as mean±standard deviation (*n*=3).

a obtained from Thailand Institute of Scientific and Technological Research.

b obtained from National Collection of Dairy Organisms, Scotland.

c obtained from National Collection of Industrial, Food and Marine Bacteria, UK.

d obtained from University College Cork culture collection.

e obtained from Chiang Mai University culture collection.

f obtained from American Type Culture Collection.

g obtained from Department of Medical Sciences Thailand.

h obtained from German Collection of Microorganisms and Cell Cultures.

### Draft Genome Sequencing and Sequence Analysis

Chromosomal DNA of strain ML122-2 was extracted using a NucleoBond^®^ kit (Macherey-Nagel, Germany). Genome sequencing of *B. velezensis* ML122-2 was performed using the Pacific Bioscience (PacBio) SMRT RSII sequencing platform (PacBio, Menlo Park, CA, United States). The obtained raw reads were assembled with the Hierarchical Genome Assembly Process (HGAP) pipeline using the protocol RS_Assembly.2 implemented in SMRT Smart Analysis portal v.2.3 (Altschul et al., 1990). Genome sequencing was also performed using an Illumina MiSeq platform by the commercial sequencing service provider Probiogenomics (University of Parma, Italy) using the chromosomal DNA of strain ML122-2, which was extracted using a PureLink^™^ Genomic DNA extraction kit according to the manufacturer’s instructions (Invitrogen^™^, CA, United States). Genomic libraries were constructed using the TruSeq DNA PCR-Free LT Kit (Illumina^®^) and 2.5μg of genomic DNA, which was fragmented with a Bioruptor NGS ultrasonicator (Diagenode, United States) followed by size evaluation using Tape Station 2,200 (Agilent Technologies, Santa Clara, CA, United States). Library samples were loaded into a Flow Cell V3 600 cycles (Illumina^®^). Fastq files of the paired-end reads (2×250bp) were used as input for genome assemblies through the MEGAnnotator pipeline in default mode (Lugli et al., 2016). Open reading frames prediction was performed by Prodigal v2.6.3 (Strepis et al., 2020). Protein-encoding genes were automatically annotated using a BlastP v2.2.26 (cut-off value of *E* 0.0001) sequence alignments against the non-redundant protein (nr) database curated by NCBI.^1^ The bacteriocin/antimicrobial gene clusters were predicted with BAGEL4 software.^2^ Meanwhile, gene clusters involved in the biosynthesis of secondary metabolites, such as those involved in the production of NRPs, and PKs, were predicted by antiSMASH software.^3^ The genome sequence was deposited in GenBank under accession number JAGTWM000000000.

### Purification and Identification of Antimicrobial Compounds in Cell Fractions

**Figure 1:**
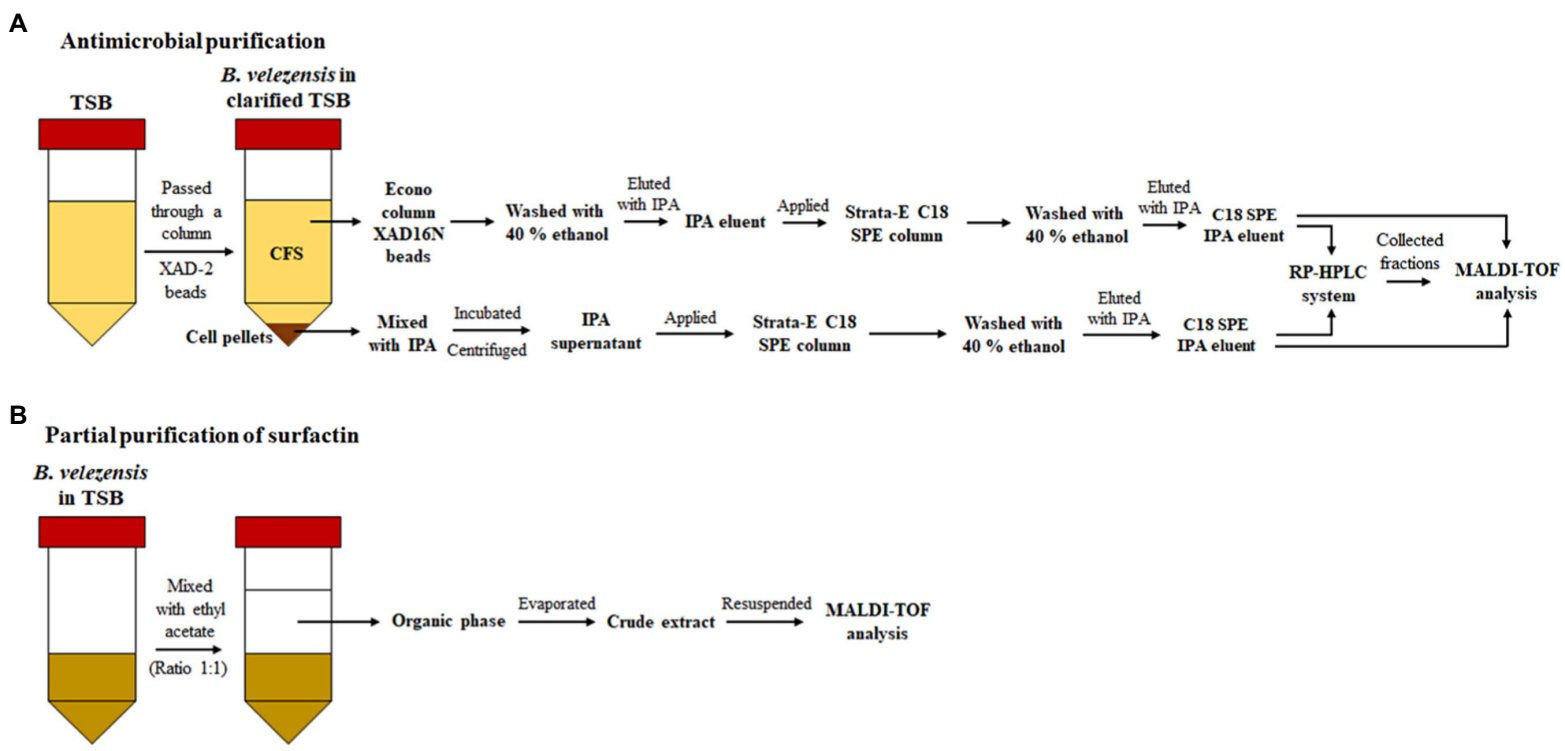
Schematic representation of antimicrobial purification by **(A)** RP-HPLC and **(B)** by solvent extraction assay (for the partial purification of surfactin).

Strain ML122-2 was cultivated in 800ml clarified TSB, which had been passed through a column containing Amberlite XAD-2 resin beads (Sigma-Aldrich^™^, St. Louis, MO, United States) to remove hydrophobic peptides, and incubated at 150rpm, 37°C for 48h prior to centrifugation at 8,000×*g* at 4°C for 20min. The resulting cell pellet was removed, and the cell-free supernatant (CFS, ~800ml) was passed through an Econo column containing 30g Amberlite^®^ XAD16N beads (Phenomenex, Cheshire, UK) prewashed with Milli Q water. Following this, the beads were washed with 250ml 40% ethanol (Fisher Scientific, UK), and bound peptides were eluted from the column with 250ml 70% (v/v) isopropanol-containing 0.1% (v/v) trifluoroacetic acid (IPA). In parallel, cells from the corresponding cell pellet were mixed with 250ml IPA and stirred at room temperature for 3–4h. Subsequently, the mixture was centrifuged at 8,000×*g* at 4°C for 20min. Both IPA eluent and IPA supernatant obtained from CFS and cell pellets, respectively (20ml each), were applied to a 1g Strata-E C18 SPE column (Phenomenex, Cheshire, UK) which was pre-equilibrated with 40% methanol and water. Each column was subsequently washed with 20ml of 40% ethanol and then eluted using 20ml IPA. The C18 SPE IPA eluents were assessed by matrix-assisted laser desorption ionization-time of flight (MALDI-TOF) mass spectrometry (Axima TOF^2^ MALDI-TOF mass spectrometer, Shimadzu Biotech, Manchester, UK) and the molecular mass of bacteriocins determined in positive ion linear mode according to the protocol described by Hill et al. (2020; Figure 1A).

### RP-HPLC Purification of Antimicrobial Peptides

Antimicrobial peptides (except surfactin, see below) were purified from CFS and cell pellets using C18 SPE and a reversed phase HPLC (RP-HPLC). The C18 SPE IPA eluents obtained as described here (Figure 1A) were applied to a semi prep Proteo Jupiter C12 (250×10mm, 4μ, 90Å) followed by running a 40 to 85% isopropanol 0.1% trifluoroacetic acid (TFA) gradient. Eluent B was 99.9% isopropanol-containing 0.1% TFA at a flow rate of 2.5ml/min. Peptide-containing fractions were detected by measuring the absorbance at 214nm. Fractions that exhibited antimicrobial activity were collected and pooled, subjected to rotary evaporation, and then lyophilized. Each purified antimicrobial peptide was resuspended in 600μl 50% isopropanol (Figure 1A). Antibacterial activity of fractions was assessed in duplicate using 6mm diameter wells, and 25μl of a given fraction/well, and employing *Bacillus cereus* TISTR 687, *B. subtilis* NCDO 10073, *Escherichia coli* DH5α, *Listeria innocua* UCC3, *Leuconostoc paramesenteroides* NCDO 869, methicillin-resistant *S. aureus* (MRSA) DMST 20625, or *S. aureus* ATCC 25923 as indicator strains. Purified antimicrobial peptides from active fractions were then subjected to MALDI-TOF MS analysis.

### Partial Purification of Surfactin From Cell-Free Supernatant

Partial purification of surfactin was achieved by organic solvent extraction according to the protocol described by Lei et al. (2020) with modifications as follows. Strain ML122-2 was cultured in TSB and incubated at 37°C, on a rotating platform at 150rpm for 48h before centrifugation at 5,000×*g* at 4°C for 15min. The supernatant was subsequently filtered through a 0.20μm nylon membrane filter. 25ml ethyl acetate (Sigma-Aldrich^™^, St. Louis, MO, United States) was mixed with 25ml filtered cell-free supernatant using a vortex mixer for 10min prior to centrifugation at 5,000×*g* at 4°C for 60min. Subsequently, the top phase (organic phase), approximately 25ml, was transferred into a glass bottle. Solvent evaporation was performed using Genevac^™^ miVac centrifugal concentrator (Genevac Limited, Suffolk, UK) at room temperature for 80min. The evaporated solvent extract was resuspended in 0.01M PBS (5ml; Figure 1B). Antibacterial activity was investigated using the agar well diffusion method described above. *B. cereus* TISTR 687, *B. subtilis* NCDO 10073, *E. coli* DH5α, *L. innocua* UCC3, *Leu. paramesenteroides* NCDO 869, MRSA DMST 20625, and *S. aureus* ATCC 25923 were used as the indicator strains. The antimicrobial-containing crude extract was then subjected to MALDI-TOF MS analysis.

### RT-qPCR Analysis

Transcriptional activity of genes associated with gene clusters predicted to be responsible for mersacidin, amylocyclicin, plipastatin, and surfactin production was investigated using RT-qPCR analysis, whereby the *mrsA*, *ancA*, *ppsA*, and *srfAA* genes served as target genes, respectively. The housekeeping gene *rpsE* was used as reference for this analysis. Primers were designed using Primer3Plus^4^ and listed in Table 2. *B. velezensis* ML122-2 was cultivated in TSB and incubated overnight at 37°C on an orbital platform shaker (150rpm) prior to centrifugation at 5000×*g* at 4°C for 10min. The resulting cell pellet was washed twice with 0.85% (w/v) NaCl and adjusted to an OD_600nm_ of 0.1. A 1% (w/w) of resuspended culture was inoculated into TSB and then incubated at 37°C at 150rpm for 48h. Cells were harvested at 24 and 48h of incubation by centrifugation at 2000×*g* for 5min. RNA extraction and cDNA synthesis were carried out using High Pure RNA Isolation Kit (Roche Diagnostics GmbH, Mannheim, Germany) and SuperScript^™^ III Reverse Transcriptase (Invitrogen^™^, CA, United States), respectively. RT-qPCR analysis of the genes or interest and reference gene were performed using a SYBR Green I Master Mix (Roche Diagnostics GmbH, Mannheim, Germany) on the LightCycler^®^ 480 II System (Roche Diagnostics GmbH, Mannheim, Germany) employing the following PCR conditions: denaturation at 95°C for 10min, followed by 40cycles of 95°C for 10s, 50°C for 15s, and 72°C for 15s. The relative expression level was calculated using the comparative 2^−(ΔΔCT)^ method (Livak and Schmittgen, 2001).

**Table 2:** Primers for RT-qPCR used in this study.

Gene target	Locus tag number	NCBI reference sequence number	Gene product	Primer (5'→3')	Reference
*mrsA*	KC480_00765	WP_224272223	Mersacidin	mrsA-F CATTCGTTCATGGAAAGATCC	This study
				mrsA-R GCCACCAGGCAATGTAAAAG	
*ancA*	KC480_04455	WP_003151973	Amylocyclicin	ancA-F GCTGCAGCAACATTGGTTTA	This study
				ancA-R TTTTTGCTGTTGCAACGATT	
*ppsA*	KC480_16150	WP_014418073	Plipastatin synthetase subunit I	ppsA-F CGCATCCATGACAGTGTACC	This study
				ppsA-R TACAGCTCGCCGAATTCTTT	
*srfAA*	KC480_07345	WP_057766256	Surfactin synthetase subunit I	srfAA-F TGACACAGAGAAGCCGAATG	This study
				srfAA-R CCAAGATCGCTAGGCGTAAG	
*rpsE*	KC480_19470	WP_003328273	30S ribosomal protein S5	rpsE-F GCGTCGTATTGACCCAAGC	Jordan et al., 2006
				rpsE-R TACCAGTACCGAATCCTACG	

## Results

### *B. velezensis* ML122-2 Exhibits a Broad Antimicrobial Range

In a previous study, *B. velezensis* (formerly *Bacillus siamensis*) ML122-2 had been demonstrated to exert antimicrobial activity against certain *S. aureus* strains (Rungsirivanich et al., 2020). Conversely, the strain did not elicit any observable antimicrobial activity against *Bacillus cereus* TISTR 687 or *E. coli* O157:H7 DMST 12743. To establish the inhibitory spectrum of this strain, the antimicrobial activity of the strain was evaluated against a panel of 23 bacterial strains and two mold species. The CFS of *B. velezensis* ML122-2 was investigated using an agar well diffusion method. *B. velezensis* ML122-2 CFS was demonstrated to inhibit 19 out of 23 assessed bacterial strains, and one out of the two molds tested in agar well diffusion assays with inhibitory/clearing zones ranging between 9.0 and 16.5mm. The CFS of *B. velezensis* ML122-2 was shown to elicit the most potent antimicrobial activity against *Leu. paramesenteroides* NCDO 869 and *P. expansum* DSM 1282 among the assessed bacteria and fungi, respectively, while it was ineffective against *B. subtilis* NCDO 1769, *B. subtilis* NCDO 10073, *E. coli* DH5α, *Levilactobacillus brevis* Rap 51, and *P. digitatum* DSM 2731 (Table 1).

### The Genome of *B. velezensis* ML122-2 Harbors Multiple Gene Clusters Associated With Antimicrobial Compound Biosynthesis

*B. velezensis* ML122-2 exhibits antimicrobial activity against *S. aureus* ATCC 25923 and MRSA DMST 20625 (Rungsirivanich et al., 2020), as well as various other microorganisms (see results above). This broad range of antimicrobial activity against a panel of microbes prompted an investigation into the nature of the antimicrobial compound(s) produced by this strain based on genome sequence analysis. To identify the antimicrobial compounds that may be produced by this strain, the genome of *B. velezensis* ML122-2 was sequenced using a combination of Illumina and PacBio sequencing technologies. The chromosome of *B. velezensis* ML122-2 was assembled into a single contig using a hybrid assembly approach employing the obtained PacBio and Illumina sequence data. This chromosomal contig consists of 4,083,790 base pairs with a 46.61% GC content, and 3,922 predicted open reading frames (ORFs). Congruently, the whole genome of *B. velezensis* ML122-2 exhibits 98.3% (94% query coverage) and 86.6% (54% query coverage) sequence identity with *B. velezensis* FZB42 (GenBank accession no. CP000560) and *B. subtilis* subsp. *subtilis* str. 168 (GenBank accession no. AL009126), respectively.

*B. velezensis* ML122-2 was previously assigned to the *B. siamensis* species based on 16S rRNA gene sequencing (Rungsirivanich et al., 2020). However, it has been suggested that *rpoB* represents a more robust marker (than the 16S rRNA gene) to determine the phylogeny of bacilli that belong to the so-called “operational group *Bacillus amyloliquefaciens,”* the latter constituting the closely related species *B. amyloliquefaciens, B. velezensis*, and *B. siamensis* (Fan et al., 2017). BlastN analysis of the *rpoB* gene of strain ML122-2 revealed 100% sequence identity with that of *Bacillus velezensis* strains and with reduced sequence identity to *rpoB* of *B. siamensis* (<98.8%) and *B. amyloliquefaciens* (<99.8%). This finding confirms that strain ML122-2 belongs to the *B. velezensis* species rather than *B. siamensis*. To validate this, the average nucleotide identity (ANI) of ML122-2 was analyzed in comparison with those of strains of the *B. amyloliquefaciens, B. siamensis*, and *B. velezensis* species. ML122-2 exhibits ANI values of 97.86, 97.85, and 94.65% with *B. velezensis* ATR2, *B. amyloliquefaciens* FBZ42, and *B. siamensis* SCSIO 05746, respectively. The ML122-2 genome was shown to lack identifiable CRISPR-Cas systems, while it is predicted to contain three prophage-associated regions (13.6, 31.8, and 28.7kb in length, respectively). Two of these appear to represent incomplete prophage regions, while one is predicted to be intact and located within positions 1,213,485-1,245,310 on the genome. This prophage region contains genes predicted to encode DNA replication enzymes, capsid and tail structural components, and lysis functions. BlastN analysis of this putative prophage region highlights that it is highly conserved among the sequenced genomes of *B. velezensis* strains.

**Figure 2:**
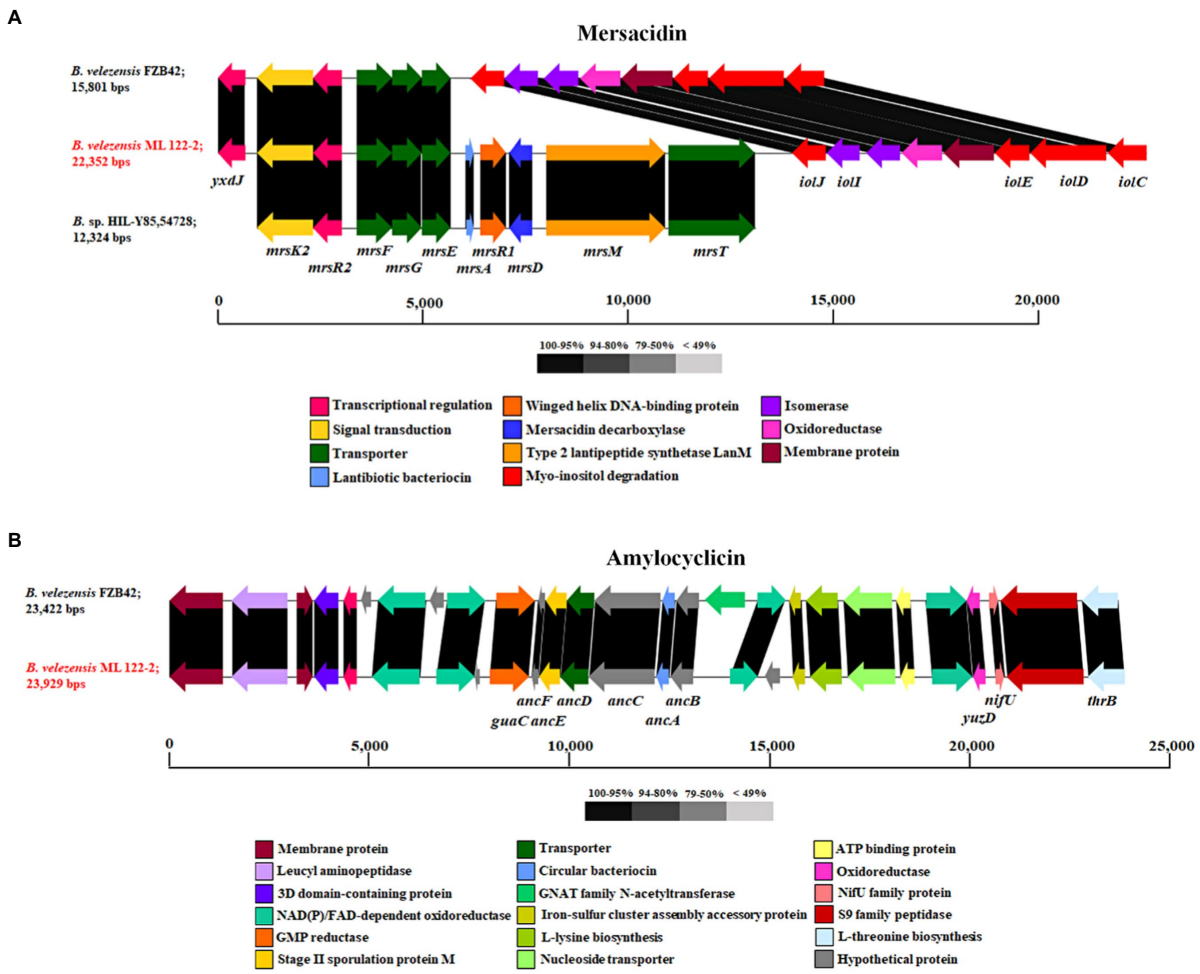
Schematic image of the gene clusters associated with the ribosomally synthesized peptides, Mersacidin **(A)** and amylocyclicin **(B)** of strains *B. velezensis* ML122-2 (red), *B. velezensis* FZB42 and *B*. sp. HIL Y-85,54,728. Predicted functions and sequence similarity are color-coded as indicated.

Further *in silico* analysis was performed using BAGEL4 and antiSMASH to identify genes involved in the production of antimicrobial or bioactive compounds. A total of four putative bacteriocin or bacteriocin-like gene clusters were predicted by BAGEL4 software including those encoding the biosynthetic and immunity genes for mersacidin, amylocyclicin, ComX, and LCI (Table 3). The predicted ML122-2 mersacidin gene cluster was shown to comprise of *mrsK2*, *mrsR2*, *mrsF*, *mrsG*, *mrsE*, *mrsA*, *mrsR1*, *mrsD*, *mrsM*, and *mrsT* and is similar to that of *Bacillus* sp. HIL-Y85/54728 (Genbank accession no. AJ250862; 98%) which was previously described by Altena et al. (2000; Figure 2A). Therefore, it appears that a complete mersacidin gene cluster is present in the *B. velezensis* ML122-2 genome. The ML122-2 genome also contains a gene cluster with high identity (98%) to the amylocyclicin cluster of *B. velezensis* FZB42 (Scholz et al., 2014; Figure 2B). The *comX* gene cluster of *B. velezensis* ML122-2 elicits 35% identity with that of *B. velezensis* FZB42 which encodes the competence pheromone ComX peptide, while the *lci* gene encodes a putative antimicrobial peptide, and exhibits 89% identity with the corresponding *lci* gene of *B. velezensis* FZB42 (Supplementary Figure 1). Gene clusters with nucleotide sequence similarity values below 30% were deemed insignificant.

**Table 3:** Identification of gene clusters involved in the ribosomally synthesized bacteriocins and secondary metabolite synthesis by *B. velezensis* ML122-2 using BAGEL4 (clusters 1, 3, 5, and 8) and antiSMASH (remainder of presented clusters).

Cluster	Genome location		Type	Bacteriocins or Secondary metabolites	Nucleotide identity (%)	Expected molecular mass (Da)	Reference
1	141,570	164,758	Lantipeptide class II	Mersacidin	98	1,826	Herzner et al., 2011
2	300,796	342,214	Other^a^	Bacilysin	100	270	Walker and Abraham, 1970
3	867,464	887,797	Class I small RiPPs	Amylocyclicin	98	6,381	Scholz et al., 2014
4	872,945	922,985	NRPS	Bacillibactin	100	882	Cheon et al., 2019
5	917,801	937,987	ComX pheromone	ComX	35	–	–
6	1,185,571	1,195,930	RiPP-like^b^	Unknown	–	–	–
7	1,401,273	1,466,079	NRPS	Surfactin	98	1,036	Gao et al., 2017
8	1,461,209	1,481,344	Bacteriocin class II	LCI	89	5,468	Zhu et al., 2001
9	1,539,768	1,616,420	TransAT-PKS^c^	Rhizocticin A	22	351	Rapp et al., 1988
10	2,183,974	2,238,601	TransAT-PKS^c^	Difficidin	46	544	Wilson et al., 1987
11	2,283,261	2,324,505	PKS-like^d^	Butirosin A/butirosin B	7	555	Dion et al., 1972
12	2,409,385	2,426,027	Terpene^e^	Unknown	–	–	–
13	2,725,345	2,813,129	TransAT-PKS^c^	Macrolactin H	100	376	Nagao et al., 2001
14	3,036,690	3,145,665	TransAT-PKS^c^	Bacillaene	97	580	Patel et al., 1995
15	3,201,379	3,339,220	TransAT-PKS^c^	Plipastatin	97	1,464	Li et al., 2012
16	3,364,156	3,386,039	Terpene^e^	Unknown	–	–	–
17	3,450,366	3,491,466	T3PKS^f^	Unknown	–	–	–
18	3,606,746	3,670,343	TransAT-PKS-like^g^	Difficidin	53	544	Wilson et al., 1987
19	4,016,677	4,080,082	TransAT-PKS-like^g^	Difficidin	26	544	Wilson et al., 1987

Secondary metabolite cluster class abbreviations according to antiSMASH.

a cluster containing a secondary metabolite-related protein that does not fit into any other category.

b other unspecified ribosomally synthesized and post-translationally modified peptide product (RiPP) cluster.

c trans-AT PKS.

d other types of PKS cluster.

e terpene.

f type III PKS.

g trans-AT PKS fragment, with trans-AT domain not found.

**Figure 3:**
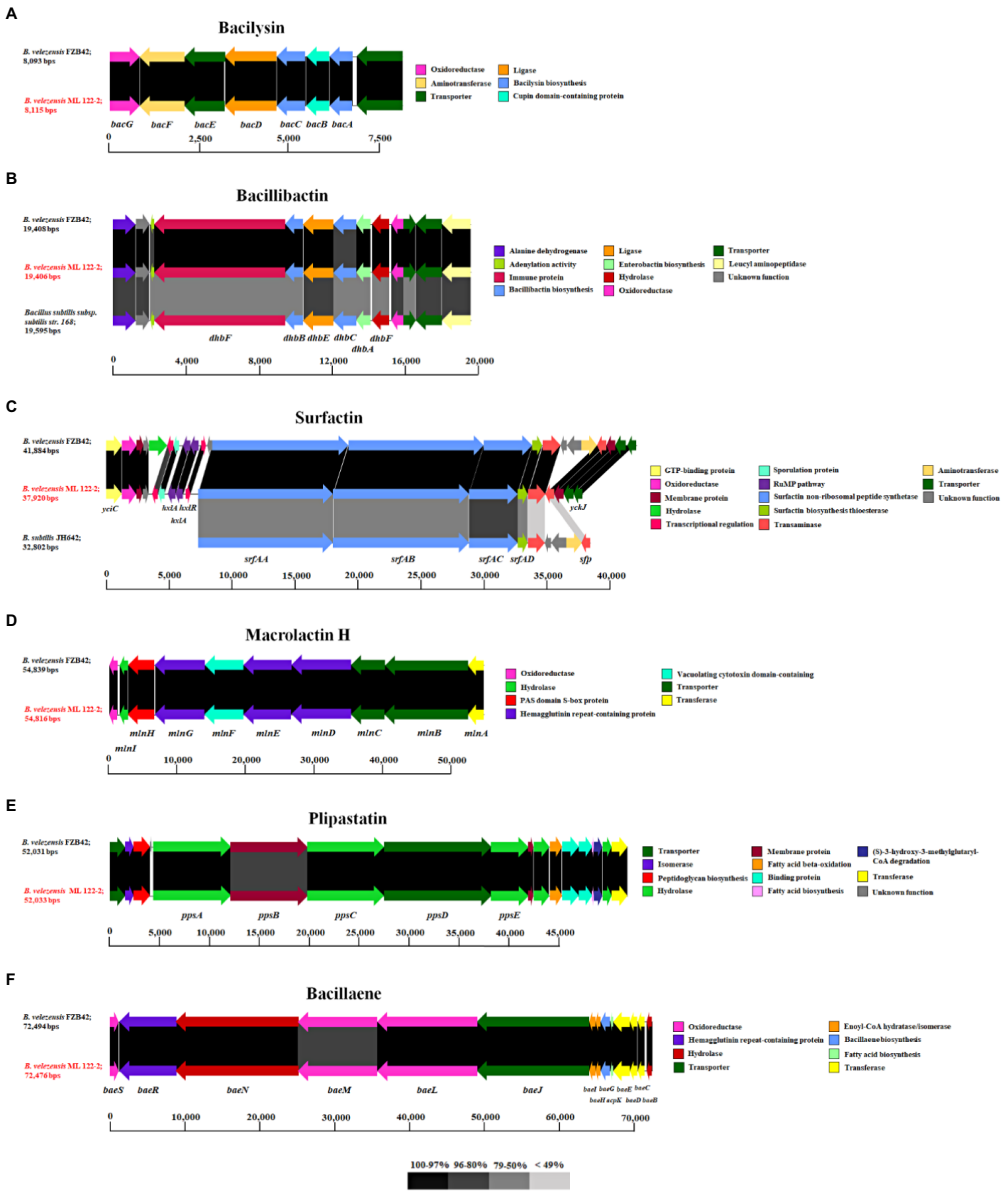
The gene clusters associated with the non-ribosomally synthesized peptides, bacilysin **(A)**, bacillibactin **(B)**, surfactin **(C)**, macrolactin H **(D)**, and plipastatin **(E)**, and polyketide, bacillaene **(F)**, of *B. velezensis* ML122-2 (red) compared to equivalent clusters in reference *Bacillus* strains. The predicted functions and sequence similarity are color-coded according to the legend.

In addition to ribosomally synthesized antimicrobial peptides, *Bacillus* spp. have been reported to produce non-ribosomally synthesized antimicrobial compounds. Sequence analysis using antiSMASH identified nine gene clusters predicted to be involved in the production of secondary metabolites including NRPs and PKs, of which six were shown to exhibit 75–100% nucleotide identity to known NRP/PK clusters from strains of *Bacillus* spp. (Table 3). Of these latter six clusters, five are predicted to encode NRPs (bacilysin, bacillibactin, surfactin, macrolactin H, and plipastatin), while the remaining one is associated with the biosynthesis of a PK (bacillaene). Genes associated with macrolactin biosynthesis are typically identified on the genomes of *B. velenzensis* strains, while they have not been observed among the genomes of *B. siamensis* or *B. amyloliquefaciens* strains (Fan et al., 2017). This finding supports the reassignment of this strain as a *B. velezensis* strain. The bacilysin- and bacillibactin-associated clusters display 100% sequence identity with equivalent clusters in *B. velezensis* FZB42 which includes seven (*bacABCDEFG*) and five (*dhbACEBF*) subunit genes (Figures 3A,B), respectively. The bacillibactin biosynthesis cluster exhibits 75% nucleotide identity with its counterpart in *B. subtilis* subsp. *subtilis* str. 168, while the surfactin gene cluster (*srfAA*, *srfAB*, *srfAC*, and *srfAD*) exhibits 98 and 79% nucleotide identity with *B. velezensis* FZB42 and *B. subtilis* JH642, respectively. The genome of *B. velezensis* ML122-2 was shown to lack the *ycxBCD* genes located downstream of the *sfp* gene (Figure 3C). Furthermore, the macrolactin H biosynthesis gene cluster, *mlnABCDEFGHI*, exhibits 100% sequence identity to those of *B. velezensis* FZB42, whereas the plipastatin biosynthesis gene cluster, *ppsABCDE*, displays 97% identity with that of *B. velezensis* FZB42 (Figures 3D,E). The bacillaene-associated gene cluster shows 97% identity to that of *B. velezensis* FZB42, which consists of eight subunit genes, *baeEDLMNJRS* (Figure 3F).

### Antimicrobial Purification and Mass Spectrometry Analysis

**Figure 4:**
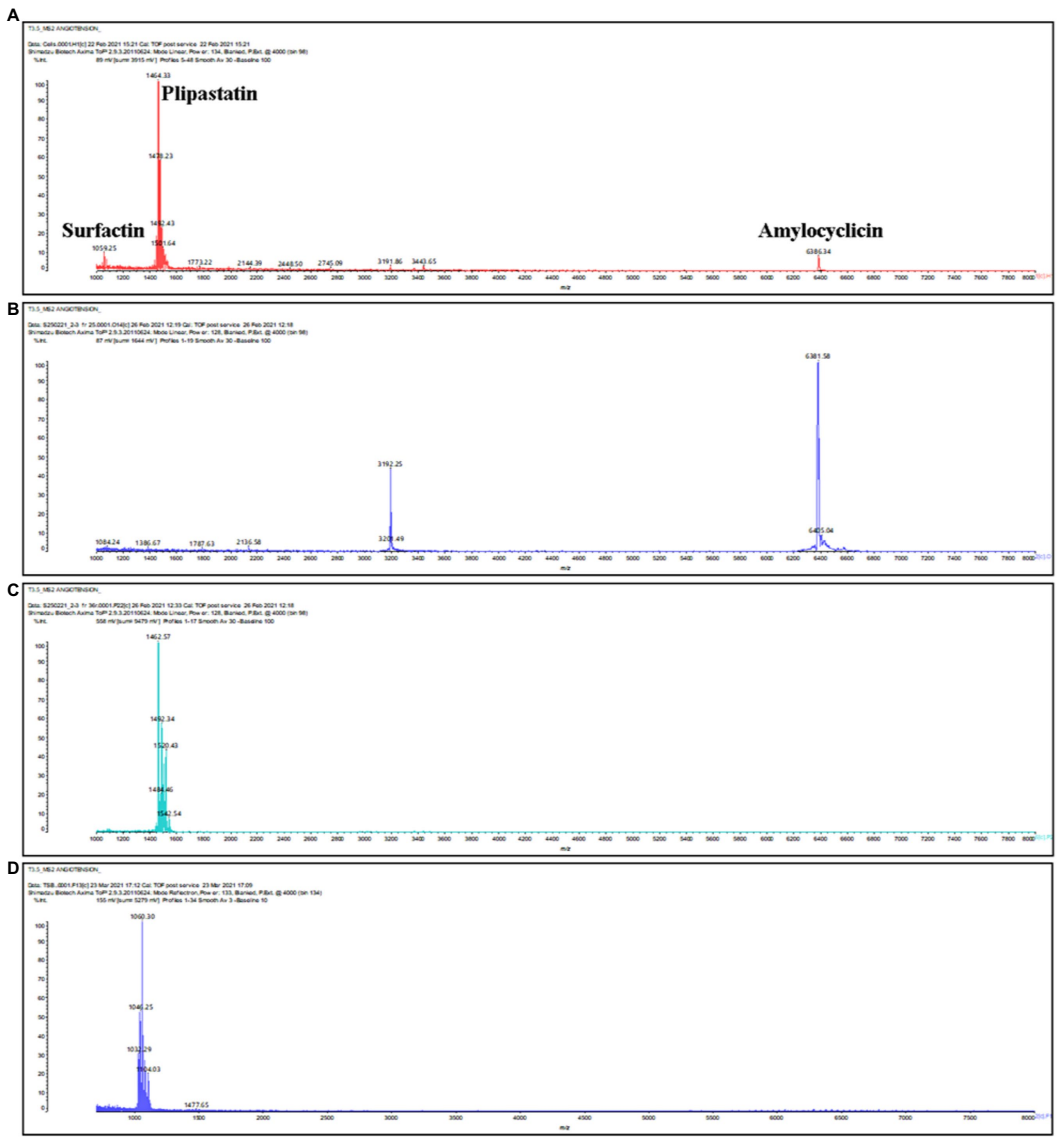
MALDI-TOF mass spectra of IPA supernatant from cell extract of *B. velezensis* ML122-2 **(A)**. Surfactin, amylocyclicin, and plipastatin were detected at *m/z* 1,059.25, 1,464.33 and 6,381.58, respectively. **(B,C)** Purification of amylocyclicin and plipastatin by RP-HPLC. The active fractions eluted at time interval of 25–28 and 31–37min in a gradient of 40–85% propan-2-ol 0.1% trifluoroacetic acid represented the peaks of purified amylocyclicin (*m/z* 6,381.58 [M+H]^+^ and 3,192.25, the doubly charged form) and plipastatin (*m/z* 1,462.57, 1,484.46 1,492.32, 1520.43 and 1542.54), respectively. **(D)** Partial surfactin purification *via* solvent extraction of *B. velezensis* ML122-2 from supernatant revealing corresponding masses at *m/z* 1,032.29, 1,046.25, 1,060.30 and 1,104.03.

Based on genome analysis, *B. velezensis* ML122-2 has the genetic capacity to produce a considerable number of distinct antimicrobial compounds. Accordingly, in order to assess which of the predicted antimicrobial compounds are responsible for the observed antimicrobial activity of *B. velezensis* ML122-2, we characterized the antimicrobial peptides produced in cell pellets and CFS extracts and analyzed the active fractions by MALDI-TOF MS (see “Materials and Methods”; Figure 1). The MALDI-TOF mass spectrum displayed major ion peaks [M+H]^+^ at *m/z* values of 1,059.25, 1,464.33, and 6,381.58, which correspond to the deduced molecular masses of surfactin (1,036kDa; Pathak et al., 2014), plipastatin (1,464kDa; Dimkić et al., 2017), and amylocyclicin (6,381kDa; Scholz et al., 2014), respectively (Table 3; Figure 4A).

Further purification by RP-HPLC allowed separation of antimicrobial activities in two active fractions, one of which corresponded to ion peaks with *m/z* values of 1,449.9, 1,463.9, 1,471.9, 1,487.9, 1,485.9, and 1,501.9 (Figure 4B), and one which corresponded to the peaks at *m/z* 6,381.4 and 3,190.3 (Figure 4C). Since surfactin could not purified by RP-HPLC, possibly due to its inherent hydrophobic nature, (partial) purification of this compound was achieved by solvent extraction with ethyl acetate. Ethyl acetate possesses a lower polarity than isopropanol, which was used in the RP-HPLC purification and may explain its (near) absence in the original purification. Moreover, a previous study revealed that surfactin extraction by ethyl acetate is associated with high purity and yield of the compound (Chen and Juang, 2008). The MALDI-TOF mass spectra of unpurified extract obtained from solvent extraction represented the peaks at *m/z* 1,032.38, 1,046.25, 1,060.24, and 1,103.99 (Figure 4D). Different molecular weights for purified plipastatin and purified surfactin have previously been described regarding the production of surfactin and fengycins/plipastatin with fatty acid side chains of 15 to 17 carbon atoms in a *Bacillus* strain (Koumoutsi et al., 2004), resulting in incremental molecular mass increases of 14Da for purified plipastatin and surfactin. A previous study by Pathak et al. (2014) reported the mass spectrum [M+H]^+^ of surfactin from *Bacillus* strain at *m*/*z* 994.7, 1,008.7, 1,022.7, 1,036.7, 1,064.7, 1,078.7, and 1,092.7 consistent with unsaturated C_12_-C_17_ β-hydroxy fatty acids. Similarly, [M+H]^+^ ions at m/z 1,433.8, 1,447.8, 1,461.8, 1,475.8, and 1,489.9 were assigned to plipastatin isoforms that correspond to unsaturated C_14_-C_18_ β-hydroxy fatty acids (Gao et al., 2017). Here, we successfully purified surfactin *via* solvent extraction. Furthermore, based on peak height, it appeared that cells represented a better source of amylocyclicin and lipopeptides than CFS (Supplementary Figure 2).

Genome analysis identified intact gene clusters associated with the biosynthesis of additional ribosomally (mersacidin) and non-ribosomally (macrolactin, bacillaene, bacilysin, and bacillibactin) synthesized compounds. However, the molecular masses associated with mersacidin, bacilysin, bacillibactin, macrolactin H, and bacillaene (Table 3) were not detected through MALDI-TOF MS in either the crude or purified extracts suggesting that these compounds are not produced under the applied laboratory conditions.

The antibacterial activity of individually purified amylocyclicin, plipastatin, and surfactin against *B. cereus* TISTR 687, *B. subtilis* NCDO 10073, *E. coli* DH5α, *L. innocua* UCC3, *Leu. paramesenteroides* NCDO 869, MRSA DMST 20625, and *S. aureus* ATCC 25923 as determined by the agar well diffusion method is presented in Table 4 and Supplementary Figure 3. The purified amylocyclicin was shown to inhibit growth of all test indicator strains with the inhibitory value ranging between 7.8 and 24.0mm, while purified plipastatin represented antimicrobial activity against *B. cereus* TISTR 687, *Leu. paramesenteroides* NCDO 869, MRSA DMST 20625, and *S. aureus* ATCC 25923, with an associated zone of inhibition ranging from 7.5 to 7.8mm, and with no inhibition observed for *B. subtilis* NCDO 10073 and *L. innocua* UCC3. The purified surfactin obtained *via* solvent extraction was demonstrated to elicit antimicrobial activity against *L. innocua* UCC 3 and *Leu. paramesenteroides* NCDO 869, producing a zone of inhibition of 11.8 and 11.7mm, respectively, whereas no inhibition was observed when *B. cereus* TISTR 687, *B. subtilis* NCDO 10073, *E. coli* DH5α, and MRSA DMST 20625 and *S. aureus* ATCC 25923 were used as indicator bacteria (Table 4; Supplementary Figure 3).

**Table 4:** Antibacterial activity of CFS, purified amylocyclicin and purified plipastatin and purified surfactin against indicator strains using an agar well diffusion assay.

Indicator strain	Zone of inhibition			
	CFS	Purified amylocyclicin	Purified plipastatin	Purified surfactin
*B. cereus* TISTR 687	10.1±0.2	7.8±0.2	7.8±0.6	0
*B. subtilis* NCDO 10073	0	10.5±0.4	0	0
*L. innocua* UCC3	11.1±0.2	19.3±0.2	0	11.8±0.2
*Leu. paramesenteroides* NCDO 869	16.5±0.0	24.0±0.4	7.8±0.2	11.7±0.5
MRSA DMST 20625	10.4±0.4	10.8±0.6	7.5±0.4	0
*S. aureus* ATCC 25923	10.4±0.4	11.8±0.2	7.5±0.4	0

Data are expressed as mean±standard deviation (*n*=3).

### Transcriptional Activity of Genes Associated With the Gene Clusters

**Figure 5:**
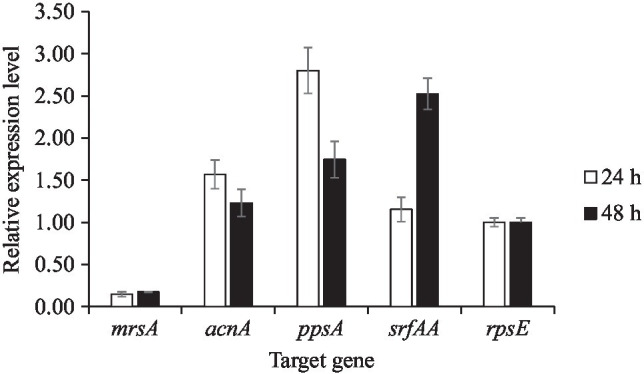
Transcriptional activity of *mrsA*, *ancA*, *ppsA*, and *srfAA* genes in 24 and 48h of *B. velezensis* cultivation. The housekeeping gene *rpsE* was used as the reference gene.

To validate the mass spectrometry-based identification of the (partially) purified compounds, transcriptional analysis of genes associated with amylocyclicin, surfactin, and plipastatin biosynthesis was undertaken. RT-qPCR analysis was performed for variation analysis of specific genes associated with amylocyclicin (*acnA*), plipastatin (*ppsA*), and surfactin (*srfAA*) production at different time points (24 and 48h) of *B. velezensis* ML122-2 cultivation. The *rpsE* gene which encodes the 30S-associated ribosomal protein S5 was used as a reference. Furthermore, since mersacidin was not detected (among others) in the analysis, it was selected as a representative negative control for the transcriptional analysis. At 24-h cultivation, *ancA*, *ppsA*, and *srfAA* genes were upregulated 1.57-, 2.80-, and 1.16-fold, while after 48-h incubation, the transcription levels were upregulated 1.23-, 1.75-, and 2.53-fold, respectively. The relative expression level of gene *mrsA* at 48-h incubation (0.18-fold) was not significant when compared with 24-h incubation (0.15-fold; Figure 5). The low transcription levels measured for the *mrsA* gene suggest lack of expression of this gene cluster, being consistent with a failure to detect mersacidin.

## Discussion

A previous study by Rungsirivanich and Thongwai (2020) revealed antibacterial activity of *B. velezensis* ML122-2 against *S. aureus* ATCC 25923 and MRSA DMST 20625 which may play a role in microbiological control strategy in Assam tea plantation areas as well as Assam tea fermentation processes. Consequently, the aim of the current study was to identify, purify, and characterize antimicrobial compounds produced by *B. velezensis* ML122-2 isolated from an Assam tea leaf. Antimicrobial activity assays revealed that various indicator bacteria are inhibited by the CFS of *B. velezensis* ML122-2. Analysis of the genome sequence of *B. velezensis* ML122-2 using BAGEL4 identified two distinct bacteriocin gene clusters including those associated with the production of mersacidin and amylocyclicin genes, while six gene clusters potentially involved in the synthesis of secondary metabolites consisting of bacilysin, bacillibactin, surfactin, macrolactin H, bacillaene, and plipastatin identified using antiSMASH. Although the genome of *B. velezensis* ML122-2 appeared to contain the complete mersacidin gene cluster, mersacidin did not appear to be synthesized by *B. velezensis* ML122-2 under laboratory conditions which is consistent with the study by Herzner et al. (2011). The failure to detect mersacidin may be due to low expression levels of *mrsA* and associated genes. Possibly, the expression of the mersacidin-associated genes may be induced under stress conditions or specific media and deserves further investigation. It has previously been shown that inactivation of *mrsR1* causes loss of mersacidin production in synthetic medium by inhibiting MrsA synthesis (Guder et al., 2002), while knockouts of *mrsR2K2* genes explicitly have been shown to prevent induction of mersacidin transcription (Schmitz et al., 2006).

Several species of *Bacillus* can produce secondary metabolites with antimicrobial activity against plant pathogenic bacteria and fungi (Lv et al., 2020), and plant growth-promoting activity with plant hormone production ability, such as indole-3-acetic acid (IAA) and 2,3-butanediol (Chen et al., 2007). Various studies have reported the production of multiple antimicrobial compounds by *Bacillus* strains. A previous study by Han et al. (2018) revealed a broad-spectrum of antimicrobial activity produced by *B. amyloliquefaciens* WY047 as a result of the simultaneous production of six antimicrobial substances. Gao et al. (2017) presented an engineered *B. subtilis* pB2-L with the ability to co-produce surfactin and plipastatin. In the current study, *B. velezensis* ML122-2 was observed to co-produce amylocyclicin, plipastatin, and surfactin. Several reports highlight the role of amylocyclicin (Chen et al., 2009), plipastatin/fengycin, and surfactin (Ongena et al., 2007) in the biological control of plant pathogens. The study by Scholz et al. (2014) revealed that amylocyclicin produced by *B. velezensis* FZB42 exhibits high antibacterial activity against Gram-positive bacteria (e.g., *B. subtilis*, *B. cereus*, *Micrococcus luteus*, and *Paenibacillus granivorans*). Surfactin exhibits antimicrobial and emulsification activities and inhibits biofilm formation (Chen et al., 2015). Plipastatin, also known as fengycin, has been reported to demonstrate antibacterial (e.g., *L. monocytogenes*, *S. aureus*, and *Salmonella* Typhimurium) and antifungal (e.g., *Fusarium oxysporum* and *Pythium ultimum*) activities causing cellular membrane distortion and cell membrane pore formation and ultimately death of cells (Gao et al., 2017; Jeong et al., 2018; Lin et al., 2020). The ability to produce multiple antimicrobial compounds has been described to increase the potential for biological control (Han et al., 2018). Our findings support the notion that *B. velezensis* ML122-2 found on Assam tea leaf plays a role in microbiological control in Assam tea or Miang cultivation *via* the production of antimicrobial peptides (Rungsirivanich et al., 2019). Consequently, *B. velezensis* that can be found both on Assam tea leaves (Rungsirivanich et al., 2020) and in fermented Assam tea products (Unban et al., 2020) may exert a powerful biocontrol function in environments, preventing food spoilage through the production of antimicrobial compounds, such as amylocyclicin, plipastatin, and surfactin.

## Conclusion

*B. velezensis* ML122-2 exhibits strong and broad-spectrum antimicrobial activity. Three antimicrobial peptides produced by *B. velezensis* ML122-2, that is, amylocyclicin, plipastatin, and surfactin, were purified from CFS and cell pellets, and their masses confirmed by MALDI-TOF mass spectrometry, this being consistent with transcriptional activity of specific marker genes for the corresponding gene clusters. Each purified peptide was shown to be antimicrobial, with amylocyclicin, in particular, eliciting substantial antimicrobial activity. These findings show that *B. velezensis* has the potential to play an important role in microbial biocontrol in Assam tea cultivation and Assam tea fermentation.

## Data Availability Statement

The datasets presented in this study can be found in online repositories. The names of the repository/repositories and accession number(s) can be found at: https://www.ncbi.nlm.nih.gov/genbank/, JAGTWM000000000, MH796212, CP000560, AL009126, AJ250862, and X70356.

## Author Contributions

PR, EP, JM, NT, and DS designed the experiments. PR, EP, PO'C, and DF analyzed the data. PR, JM, and DS investigated the data. NT and DS acquired the funding. PR and NT prepared the original draft. PR, PO'C, JM, NT, and DS reviewed and edited the manuscript. All authors contributed to the article and approved the submitted version.

## Funding

This research was funded by the National Research Council of Thailand, grant number PHD60I0089; Tea Gallery Group (Thailand) Co., Ltd.; Amazing Tea Limited Partnership; the scholarship for Teaching Assistant and Research Assistant (TA/RA) awarded by the Graduate School, Chiang Mai University, Thailand and the APC Microbiome Ireland, University College Cork, Ireland. This publication has emanated from research conducted with the financial support of Science Foundation Ireland under Grant numbers 15/SIRG/3430, SFI/12/RC/2273-P1, and SFI/12/RC/2273-P2.

## Conflict of Interest

The authors declare this study received funding from Tea Gallery Group (Thailand) Co., Ltd. and Amazing Tea Limited Partnership. The funders were not involved in the study design, collection, analysis, interpretation of data, the writing of this article, or the decision to submit it for publication. All authors declare no other competing interests.

## Publisher’s Note

All claims expressed in this article are solely those of the authors and do not necessarily represent those of their affiliated organizations, or those of the publisher, the editors and the reviewers. Any product that may be evaluated in this article, or claim that may be made by its manufacturer, is not guaranteed or endorsed by the publisher.

## Footnotes

^1^ ftp://ftp.ncbi.nih.gov/blast/db/

^2^ http://bagel4.molgenrug.nl/

^3^ https://antismash.secondarymetabolites.org

^4^ https://www.bioinformatics.nl/cgi-bin/primer3plus/primer3plus.cgi
