# Supplementary material for: Simultaneous Production of Multiple Antimicrobial Compounds by Bacillus velezensis ML122-2 Isolated From Assam Tea Leaf [Camellia sinensis var. assamica (J.W.Mast.) Kitam.]
Source: Front Microbiol. 2021 Nov 24;12:789362. doi: 10.3389/fmicb.2021.789362 (PMC8653701; doi:10.3389/fmicb.2021.789362)
Supplement: Supplementary file 1 [file Data_Sheet_1.PDF]

Supplementary Material

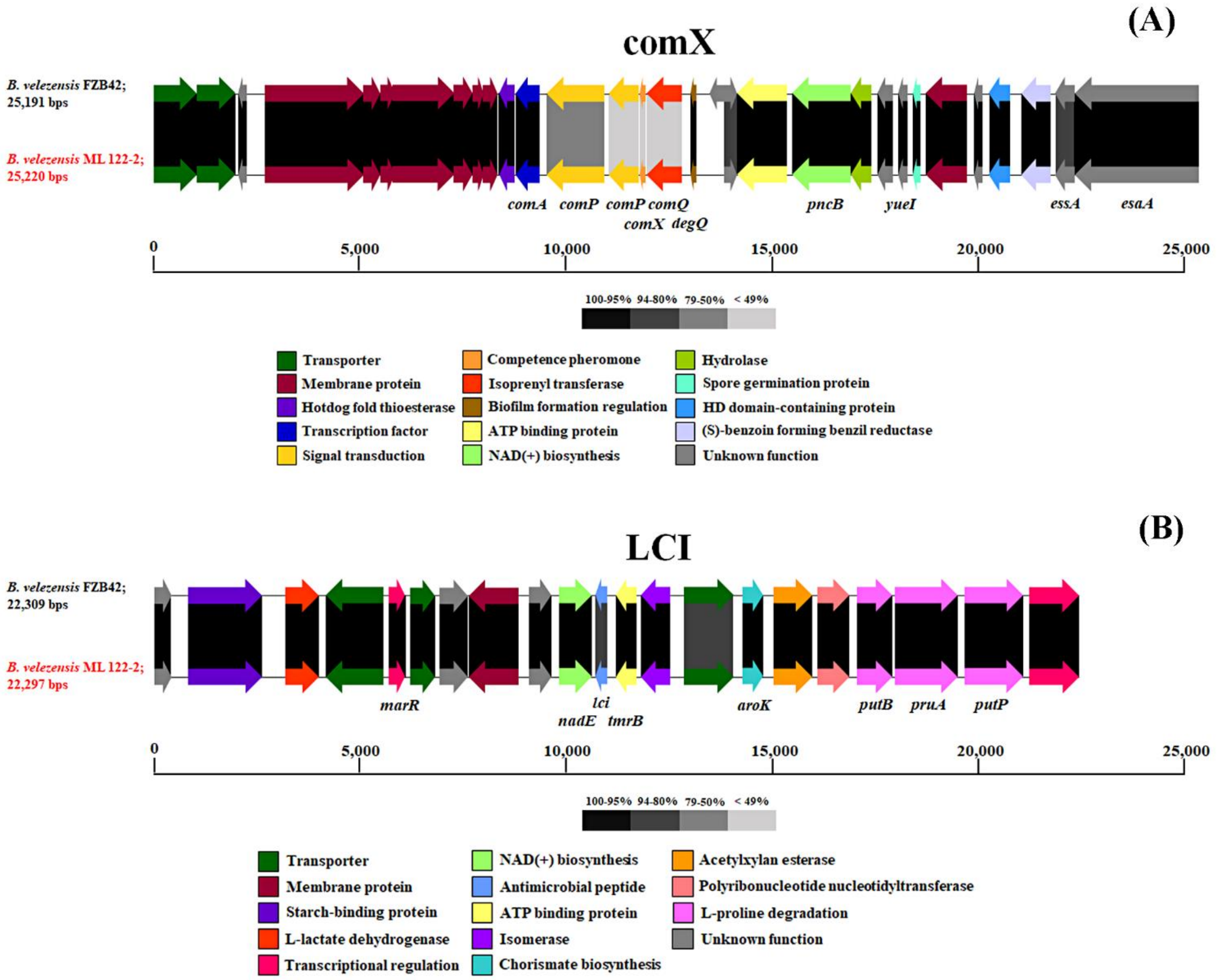

Supplementary Figure 1. The comX **(A)** and LCI **(B)** gene clusters of *B. velezensis* ML122-2 (red) compared with *B. velezensis* FZB42 (black).

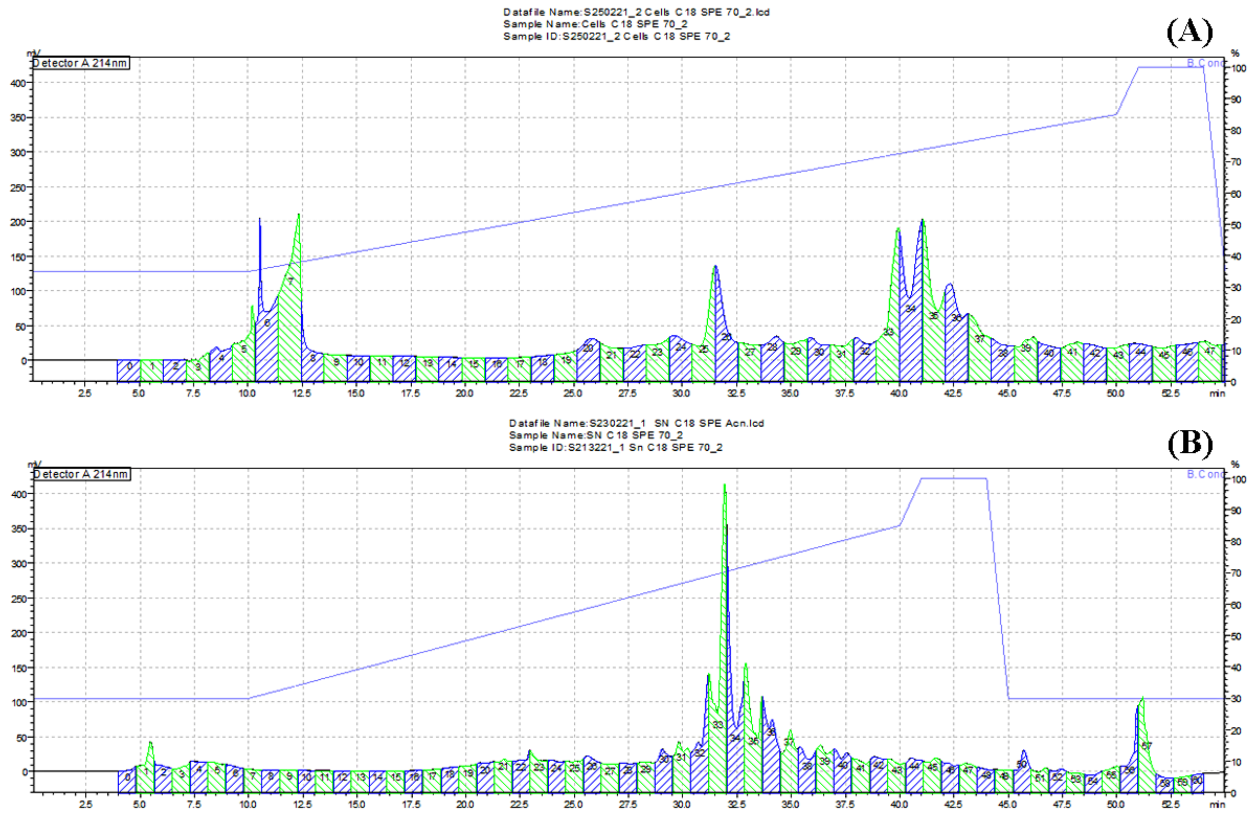

Supplementary Figure 2. Antimicrobial peptides purification of *B. velezensis* ML122-2 by RP-HPLC system. The C18 SPE IPA eluents obtained from cell pellets extract **(A)** and cell-free supernatant **(B)** were applied to a semi prep Proteo Jupiter C12 (250 x 10 mm, 4µ, 90Å) followed by running a 40 to 85 % isopropanol 0.1 % trifluoroacetic acid (TFA) gradient. Eluent B was 99.9 % isopropanol containing 0.1 % TFA at a flow rate of 2.5 ml/min. The peaks were detected measuring the absorbance at 214 nm and the fractions were collected at 1 minute intervals.

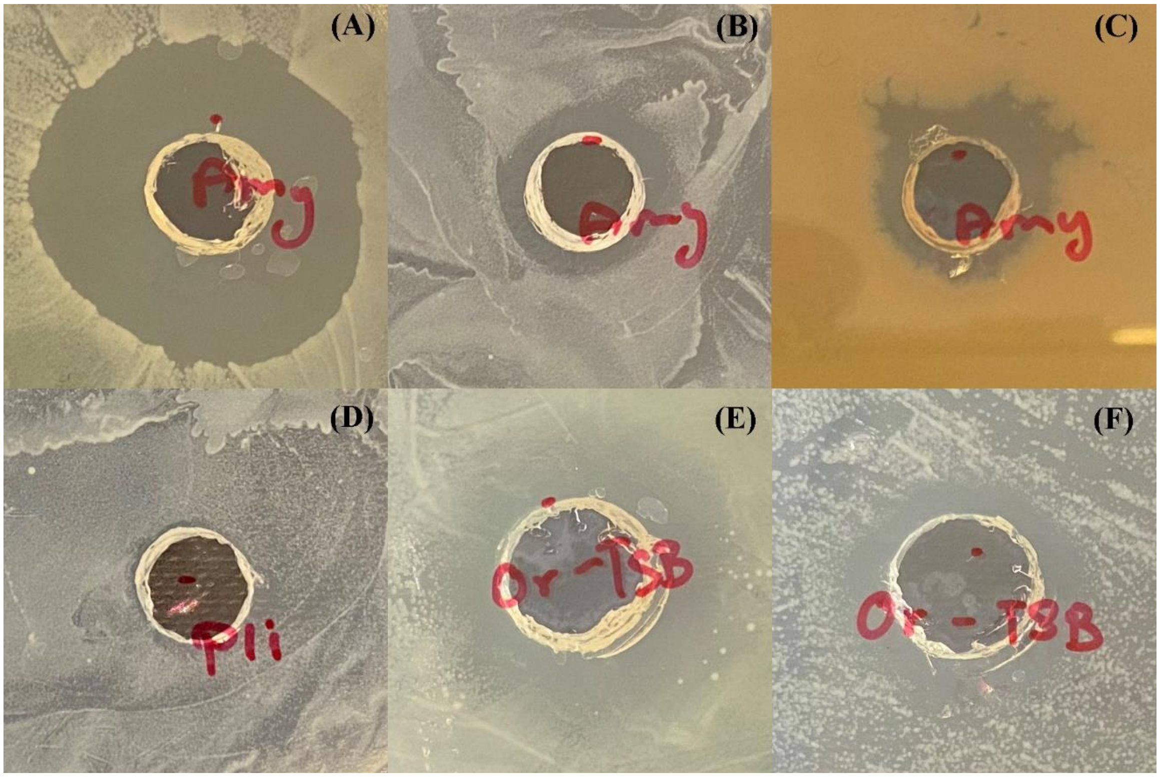

Supplementary Figure 3. Antibacterial activity of purified amylocyclicin against *Listeria innocua* UCC3 **(A)**, methicillin resistant *Staphylococcus aureus* (MRSA) DMST 20625 **(B)** and *B.* *subtilis* NCDO 10073 **(C)**, purified plipastatin against MRSA DMST 20625 **(D)** and purified surfactin against *L. innocua* UCC3 **(E)** and *Leu. paramesenteroides* NCDO 869 **(F)**.
